# Human and entomologic investigations of chikungunya outbreak in Mandera, Northeastern Kenya, 2016

**DOI:** 10.1371/journal.pone.0205058

**Published:** 2018-10-11

**Authors:** Samson Limbaso Konongoi, Albert Nyunja, Victor Ofula, Samuel Owaka, Hellen Koka, Edith Koskei, Fredrick Eyase, Daniel Langat, James Mancuso, Joel Lutomiah, Rosemary Sang

**Affiliations:** 1 Kenya Medical Research Institute, Nairobi, Kenya; 2 United States Army Medical Research Directorate, Nairobi, Kenya; 3 Jomo Kenyatta University of Agriculture and Technology, Nairobi, Kenya; 4 Kenya Ministry of Health -Division of Disease Surveillance and Response, Nairobi, Kenya; Singapore Immunology Network, SINGAPORE

## Abstract

Chikungunya is a reemerging vector borne pathogen associated with severe morbidity in affected populations. Lamu, along the Kenyan coast was affected by a major chikungunya outbreak in 2004. Twelve years later, we report on entomologic investigations and laboratory confirmed chikungunya cases in northeastern Kenya. Patient blood samples were received at the Kenya Medical Research Institute (KEMRI) viral hemorrhagic fever laboratory and the immunoglobulin M enzyme linked immunosorbent assay (IgM ELISA) was used to test for the presence of IgM antibodies against chikungunya and dengue. Reverse transcription polymerase chain reaction (RT-PCR) utilizing flavivirus, alphavirus and chikungunya specific primers were used to detect acute infections and representative PCR positive samples sequenced to confirm the circulating strain. Immature mosquitoes were collected from water-holding containers indoors and outdoors in the affected areas in northeastern Kenya. A total of 189 human samples were tested; 126 from Kenya and 63 from Somalia. 52.9% (100/189) tested positive for Chikungunya virus (CHIKV) by either IgM ELISA or RT-PCR. Sequence analysis of selected samples revealed that the virus was closely related to that from China (2010). 29% (55/189) of the samples, almost all from northeastern Kenya or with a history of travel to northern Kenya, tested positive for dengue IgM antibodies. Entomologic risk assessment revealed high house, container and Breteau indices of, 14.5, 41.9 and 17.1% respectively. Underground water storage tanks were the most abundant, 30.1%, of which 77.4% were infested with *Aedes aegypti* mosquitoes. These findings confirm the presence of active chikungunya infections in the northeastern parts of Kenya. The detection of dengue IgM antibodies concurrently with chikungunya virus circulation emphasizes on the need for improved surveillance systems and diagnostic algorithms with the capacity to capture multiple causes of arbovirus infections as these two viruses share common vectors and eco-systems. In addition sustained entomological surveillance and vector control programs targeting most productive containers are needed to monitor changes in vector densities, for early detection of the viruses and initiate vector control efforts to prevent possible outbreaks.

## Background

With its origins in Africa and its discovery in 1952 along the Tanzania Mozambique border, chikungunya virus (CHIKV) is a re-emerging vector borne alphavirus associated with human illnesses in various parts of Africa and Asia. It is associated with outbreaks causing severe disease morbidity among susceptible populations with the potential for persistent arthralgia and long-term impaired physical functionality on the victims [1]. Previously, chikungunya was associated with non-life-threatening infections, but all this changed when an outbreak accompanied with mother to child transmission, neurological and hemorrhagic manifestations and mortality was reported in La Reunion in 2006 [2,3].

Four CHIKV genotypes have been documented; in sub-Sharan Africa the East-Central-South-African (ECSA) and West African genotypes are endemic and associated with epidemics while the Asian genotype and the Indian Ocean lineage (IOL) are associated with epidemics in parts of Asia and the Indian Ocean islands [4].

Chikungunya virus is maintained in enzootic cycles among non-human primates and is transmitted by *Aedes* mosquito species, with human infections occurring as a result of the bite of infected mosquitoes [5]. In Africa, CHIKV is maintained during inter epidemic periods in a sylvatic transmission cycle involving a number of species of mosquitoes including: *Ae*. *aegypti*, *Ae*. *africanus*, *Ae*. *luteocephalus* and *Ae*. *furcifer-taylori*, and non-human primates. It’s been isolated from these mosquito species in multiple African countries with diverse geographical and ecological conditions. In Asia, the virus is maintained in cycles between *Ae*. *aegypti* or *Ae*. *albopictus* and humans [6]. These vectors are widely distributed in many parts of the world including Europe and the Americas [7, 8]. Prior to the outbreaks in La Reunion and Indian Ocean Islands in 2005–2006; *Ae*. *aegypti* was considered to be the main vector associated with CHIKV transmission. However, in the La Reunion outbreak, *Ae*. *albopictus* was identified as the main transmission vector and this was attributed to a single mutation in the envelope (E1) protein gene of the circulating strains of Indian lineage, which had evolved from the ECSA enzootic lineage. *Ae*. *albopictus* is widely distributed in various European and Mediterranean countries, posing a risk of introduction of chikungunya as a result of this mutation [9].

The first major outbreaks associated with chikungunya occurred in Thailand and India in the 1960’s and 70s mainly in urban settings [10]. In the last 3 decades, no other major outbreak was reported worldwide until 2004 when a large outbreak occurred in Lamu along the Kenyan coast with an estimated 13,500 cases detected [11]. By 2005, the outbreak had spread to the Indian Ocean Islands of the Comoros and La Reunion with over 500,000 cases being reported [12, 13]. Within 4 years, the outbreak had spread from the African continent to parts of South East Asia and India affecting millions of people [14]. Rapid spread of the virus has been associated with commerce and global connectivity. Imported cases have been documented in multiple countries throughout Europe, Asia and North America far from the original outbreak points [10, 15–16]. Outbreaks associated with chikungunya have been shown to spread rapidly and have severe impacts on public health in affected countries [13].

The town of Mandera in northeastern Kenya had been at the center of an ongoing dengue outbreak since 2011 caused mainly by dengue virus 2 and 3 and is in close proximity to Somalia where dengue cases have been known to occur [17]. Chikungunya and dengue infections are known to occur in similar geographic areas and share a common principal vector; *Aedes* mosquito species. Co-infections have been shown to occur in 13 of 98 countries where both viruses are endemic. CHIKV infections are often confused with dengue as both infections may present with febrile manifestations and myalgias, disease symptoms that are difficult to distinguish especially if diagnosis is symptom based and not laboratory confirmed. This therefore presents a challenge in disease detection in affected populations [18]. The clinical symptoms of CHIKV infection often mimic those of dengue fever and because CHIKV circulates in regions where dengue virus is endemic, it has been postulated that many cases of dengue virus infection are misdiagnosed and that the incidence of CHIKV infection is much higher than reported [19].

## Materials and methods

### Ethics statement

We are reporting data on anonymized samples. Consent was not obtained as this was a non-research public health response activity where samples were collected at the respective hospitals and referred to KEMRI for testing. Scientific and ethical approval to share this data was obtained from the Kenya Medical Research Unit Scientific and Ethics Review unit (protocol KEMRI/RES/7/3/1) approving the dissemination of data generated anonymously to the public health and scientific communities through publications.

**Fig 1:**
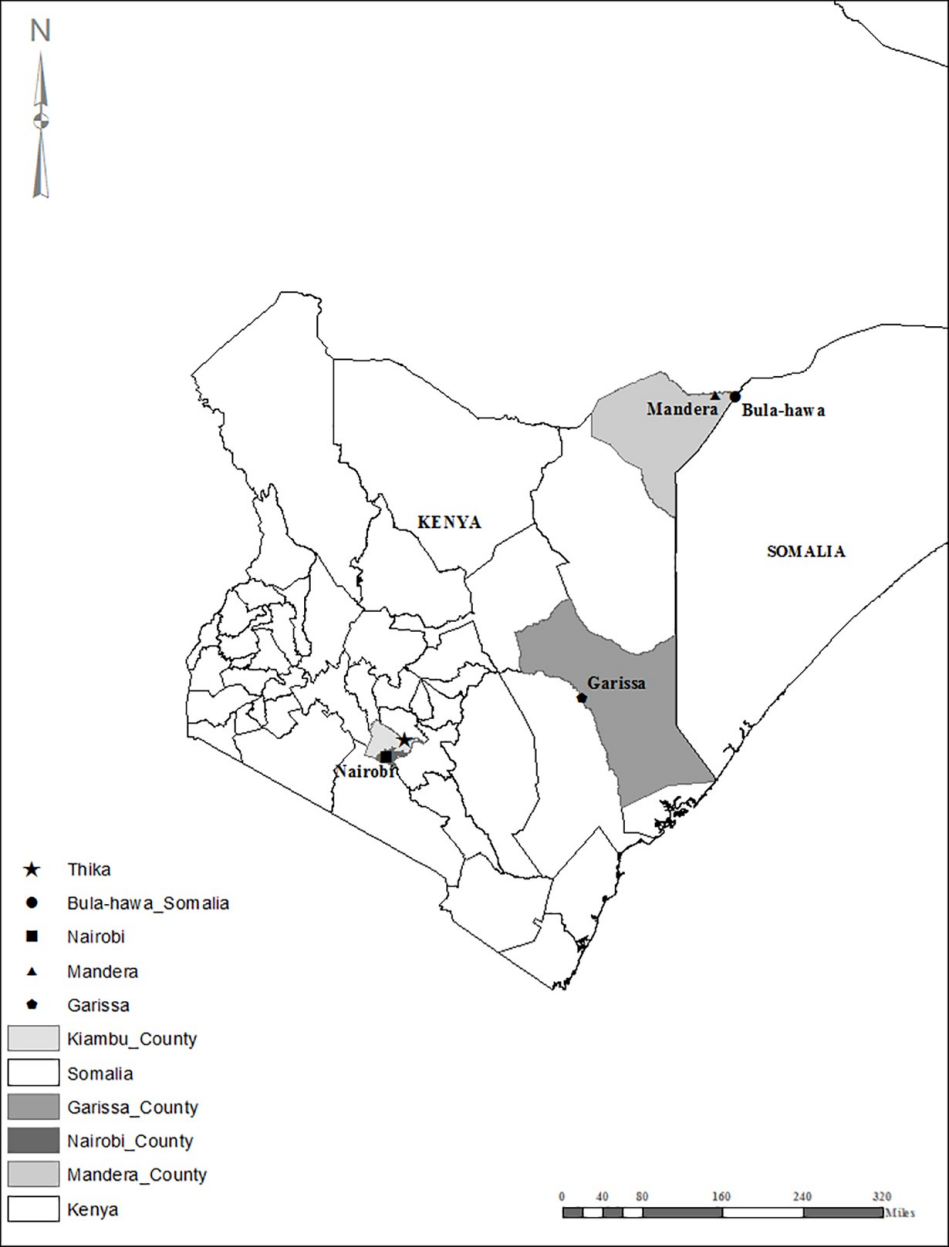
Map of Kenya showing regions from which human samples were obtained for testing.

In May 2016 there were reports of an increase in human cases with manifestations of fever and joint pains in Mandera East sub-county near the borders of Somalia and Ethiopia. Samples were collected from all subjects presenting with these clinical signs regardless of age or sex. In addition, entomological investigations were carried out in the affected areas in June 2016 (Fig 1).

### Human sample collection and testing

Using standard phlebotomy techniques, venous blood was collected in vacutainer tubes with no anticoagulant at the respective health facilities and transported in cold chain to the KEMRI laboratory in Nairobi. The blood was centrifuged to obtain serum for laboratory testing. The IgM antibody capture enzyme-linked immunosorbent assay (MAC-ELISA) was used to detect IgM antibodies against chikungunya and Dengue. Alphavirus and flavivirus family and chikungunya specific RT-PCR primers were used to detect acute infections. A subset of chikungunya RT-PCR positive samples all from Mandera; were sequenced to confirm their identity and determine the relationship of the infecting virus to others in the gene bank.

### Mosquito collection

All water-holding containers found indoors (inside every accessible house) and outdoors (immediately outside the inspected houses and within the peridomestic environment) were inspected for immature mosquitoes. Samples from each positive container were collected using ladles and pipettes or, in the case of jerry cans, the water was poured through a fine sieve onto a white basin and the larvae or pupae then picked from the sieve using pasture pipettes. The collections were geocoded using Global Positioning System (GPS) to the premises where they were collected. Live immature mosquitoes sampled from each water container type were reared to adults and morphologically identified to species using taxonomic keys [20]. Indoor and outdoor containers were then scored separately as either being wet negative (with no *Ae*. *aegypti* immatures) or wet positive (with at least one immature *Ae*. *aegypti*).

### Larval indices

The mosquito larval indices were calculated as House Index (HI)—the percentage of houses surveyed that were positive with immature *Ae*. *aegypti* mosquitoes, Container Index (CI)—the percentage of containers with water that were found infested with immature *Ae*. *aegypti* mosquitoes and Breteau Index (BI)—the number of containers positive for immature *Ae*. *aegypti* mosquitoes per 100 houses sampled [21]. The following formulas were used to determine these indices:

$$\begin{array}{l} \mathrm{HI}=\frac{\mathrm{Number}\;\mathrm{of}\;\mathrm{houses}\;\mathrm{with}\;\mathrm{immature}\;\mathrm{mosquitoes}}{\mathrm{Number}\;\mathrm{of}\;\mathrm{inspected}\;\mathrm{houses}}X\;100\\ \mathrm{CI}=\frac{\mathrm{Number}\;\mathrm{of}\;\mathrm{containers}\;\mathrm{with}\;\mathrm{active}\;\mathrm{breeding}}{\mathrm{Number}\;\mathrm{of}\;\mathrm{wet}\;\mathrm{containers}}X\;100\\ \mathrm{BI}=\frac{\mathrm{Number}\;\mathrm{of}\;\mathrm{positive}\;\mathrm{containers}}{\mathrm{Number}\;\mathrm{of}\;\mathrm{inspected}\;\mathrm{houses}}X\;100 \end{array}$$

## Laboratory analysis

### MAC-enzyme linked immunosorbent assay

A laboratory derived IgM antibody capture ELISA (MAC-ELISA) provided by the Diagnostic Systems Division, United States Army Medical Research Institute of Infectious Diseases (USAMRIID) was used to test for the presence of chikungunya IgM antibodies. Commercial anti-human IgM antibody (goat anti-human IgM, Kirkegaard and Perry laboratories Gaithersburg, MD, USA) was coated on a 96 well Immunolon plate (Nunc, Denmark) and incubated at 4°C for 12–16 hours. The plate was then washed using a wash buffer (PBS, pH 7.4, 0.01 Merthiolate, 0.1 Tween-20). This was followed by addition of the chikungunya IgM positive control, negative control and sample all diluted to 1:100 in diluent buffer (PBS, pH 7.4, 0.01 Merthiolate, 0.1 Tween-20, 5% skim milk). The plates were incubated at 37°C for one hour, washed and 100 μl of chikungunya antigen solution was added in one half of the test wells and a corresponding negative antigen (same dilution) was added in the other half of the test wells and incubated for one hour at 37°C.

ELISA plates were washed and 100 μl of Chikungunya specific detector antibodies (anti-chikungunya hyperimmune mouse ascitic fluid) added to each well and incubated for one hour at 37°C. The plate was washed and 100 μl of Horse Radish Peroxidase (HRP) labelled goat anti-mouse IgG, conjugate (Kirkergard and Perry, catalog 074–1806) added in all the wells and incubated for one hour at 37°C. The plate was then washed and 100 μl of ABTS substrate (Kirkergard and Perry, Cat. No. N8 50-62-00, Gaithersburg, MD) was added and incubated at 37°C for 30 minutes. The reaction was visualized by a green colour and the optical density (OD value) was read with a spectrophotometer at 405nm. The adjusted OD was calculated by subtracting the OD of the negative/mock antigen coated wells from the positive antigen coated wells. The OD cut-off was calculated as the mean of the adjusted OD of the negative control sera plus three times the standard deviations.

All the samples were tested for dengue IgM antibodies using the Novatec Immunodiagnostica dengue virus IgM ELISA kit following the manufacturer’s instructions. Briefly, 100μl of appropriately diluted test specimens and controls were dispensed into the respective wells as outlined in the kit protocol and incubated for 1 hour at 37°C. The wells were then washed 3 times and 100μl of dengue virus anti-IgM Conjugate was dispensed into all wells, except for the blank well, and incubated at room temperature for 30 minutes. The plates were washed 3 times and 100μl of Tetramethylbenzidine (TMB) substrate solution was dispensed into all wells and incubated for 15 minutes at room temperature in the dark. 100μl of stop solution was dispensed in all the wells and absorbance of the specimen measured using an ELx800 absorbance microplate reader (Biotek Winooski, Vermont, USA) at 450/620 nm. Cut off values were then calculated as per the kit instructions and positive samples identified.

### Nucleic acid (RNA) extraction

The QIAamp Viral RNA Minikit (QIAGEN, Hilden Germany) was used to extract viral RNA according to the manufacture’s protocol with 60 μl of RNA being obtained for subsequent complementary DNA (cDNA) synthesis.

### cDNA synthesis from viral RNA

In a 200 μl PCR tube, 10 μl of the extracted sample RNA was mixed with 2 μl of 50 ng/μl random hexamer primer and 1 μl of 10 mM deoxynucleotide solution (dNTPs), and incubated in a thermocycler for 5 minutes at 65°C and immediately chilled for 1 minute at 4°C. The following components were then added to the PCR tube: 4 μl of 5X First Strand Buffer (Invitrogen), 1 μl of 0.1 M DTT, 1 μl of RNAse OUT(40 U/μl) and 1 μl of Superscript III Reverse transcriptase (200 U/μl). The mixture was then incubated in a thermocycler for 5 minutes at 25°C, 50 minutes at 50°C and 15 minutes at 70°C. A total of 20 μl cDNA was obtained.

### PCR amplification

The PCR amplification of targeted viral sequences in the cDNA was performed in a 25-μL reaction containing: 12.5 μl of Amplitaq Gold 360 PCR master mix (Applied Biosystems USA), 0.5μl of 50 picomoles each of forward and reverse primer, 2 μl of the cDNA and 9.5 μl of DEPC treated water. Samples were first tested using alphavirus family primers (VIR2052F 5'-TGG CGC TAT GAT GAA ATC TGG AAT GTT-3' and VIR2052R 5'-TAC GAT GTT GTC GTC GCC GAT GAA-3') [22] and flavivirus family primers (FU1 5'- TAC AAC ATG ATG GGA AAG AGA GAG AA-3' and CFD2 5'- GTG TCC CAG CCG GCG GTG TCA TCA GC-3') [23]. Samples testing positive with alphavirus family primers were further tested with chikungunya specific primers (7028 forward (5'-TGCGCGGCCTTCATCGGCGACTAC-3') and 8288 reverse (5'-CCAGGTCACCACCGAGAGGG-3')) [24].

In all the PCR reactions, appropriate positive control cDNA and a negative control were included. Electrophoresis of the amplified DNA products was done on a 1–2% agarose gel in 1% Tris-borate EDTA buffer stained with ethidium bromide. An ultra-violet (UV) trans illuminator was used to visualize the PCR product bands and images recorded using a gel photo imaging system.

### Sequencing and phylogenetic analysis

Amplicons were purified using Wizard SV Gel and PCR Clean-Up System kit (Promega Madison, WI, USA) and sequenced using the ABI-PRISM 3130 Genetic Analyzer (Applied Biosystems, Foster City, CA). Raw chromatogram files for both forward and reverse sequences were edited for bad calls in Chromas version 2.4.4, Basic Local Alignment Search Tool was used to search the Gene Bank and confirm identity of the virus isolates. Sequence alignment was accomplished using the Muscle algorithm. The phylogenetic tree was inferred using the Maximum Likelihood method based on the General Time Reversible Model [25] (10000 bootstraps) in Molecular Evolutionary Genetics Analysis (MEGA) version 7.0[26].

## Results

**Fig 2:**
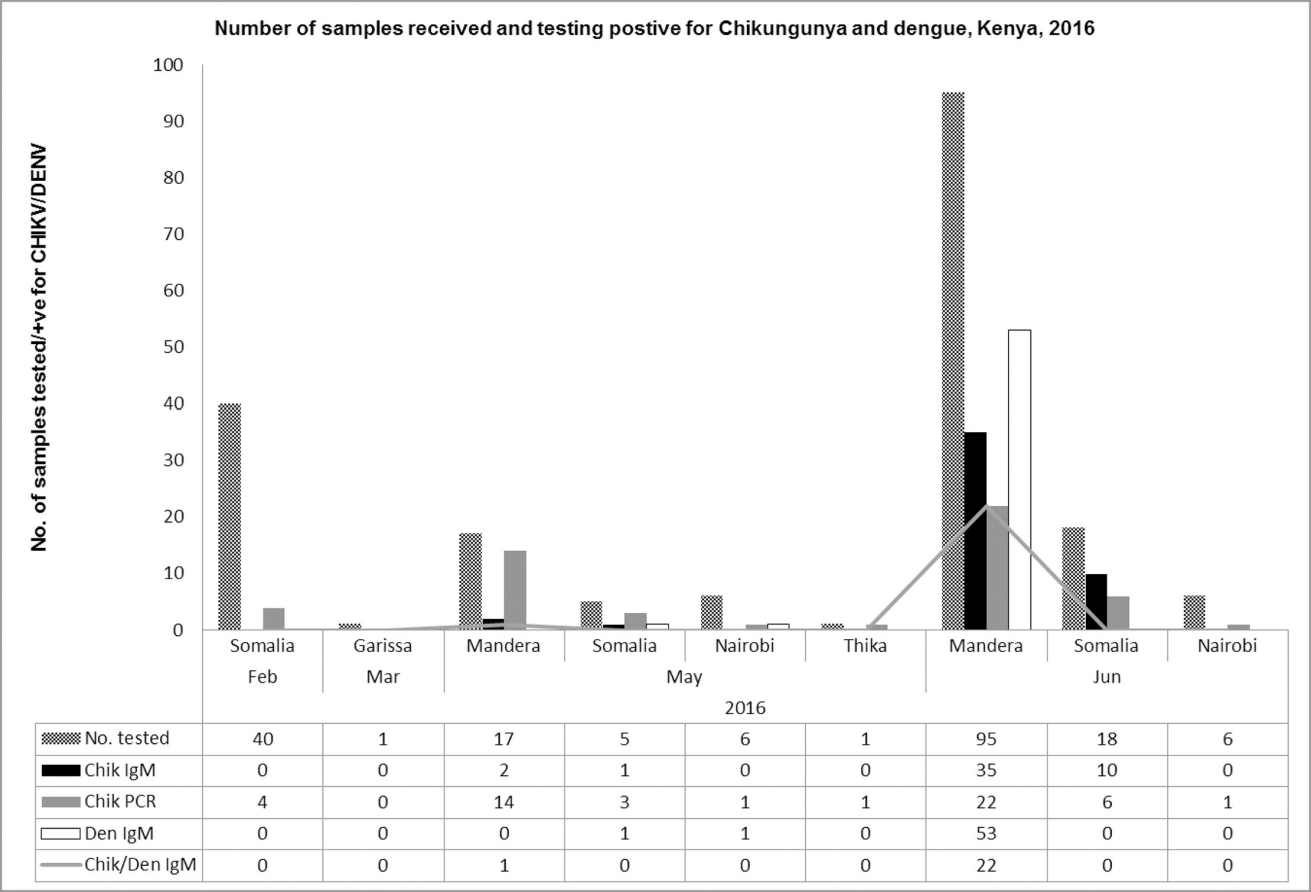
Graph showing the number of samples received and the number positive for Chikungunya and Dengue, February–June 2016.

From February to June 2016, 189 samples were received from Kenya (126) and Somalia (63) (Fig 1). 60.7% (113/189) of the samples were received from northeastern Kenya while the rest were received from Somalia and other towns in Kenya. 52.9% (100/189) tested positive for CHIKV by either IgM ELISA or RT-PCR. 29% (55/189) of the samples tested positive for dengue IgM antibodies with 12.2% (23/189) of all the samples being positive for both dengue and chikungunya IgM antibodies. Out of the 55 samples that were positive for dengue IgM antibodies, 16 were positive for chikungunya by RT-PCR. No dengue cases were detected by RT-PCR (Fig 2).

**Fig 3:**
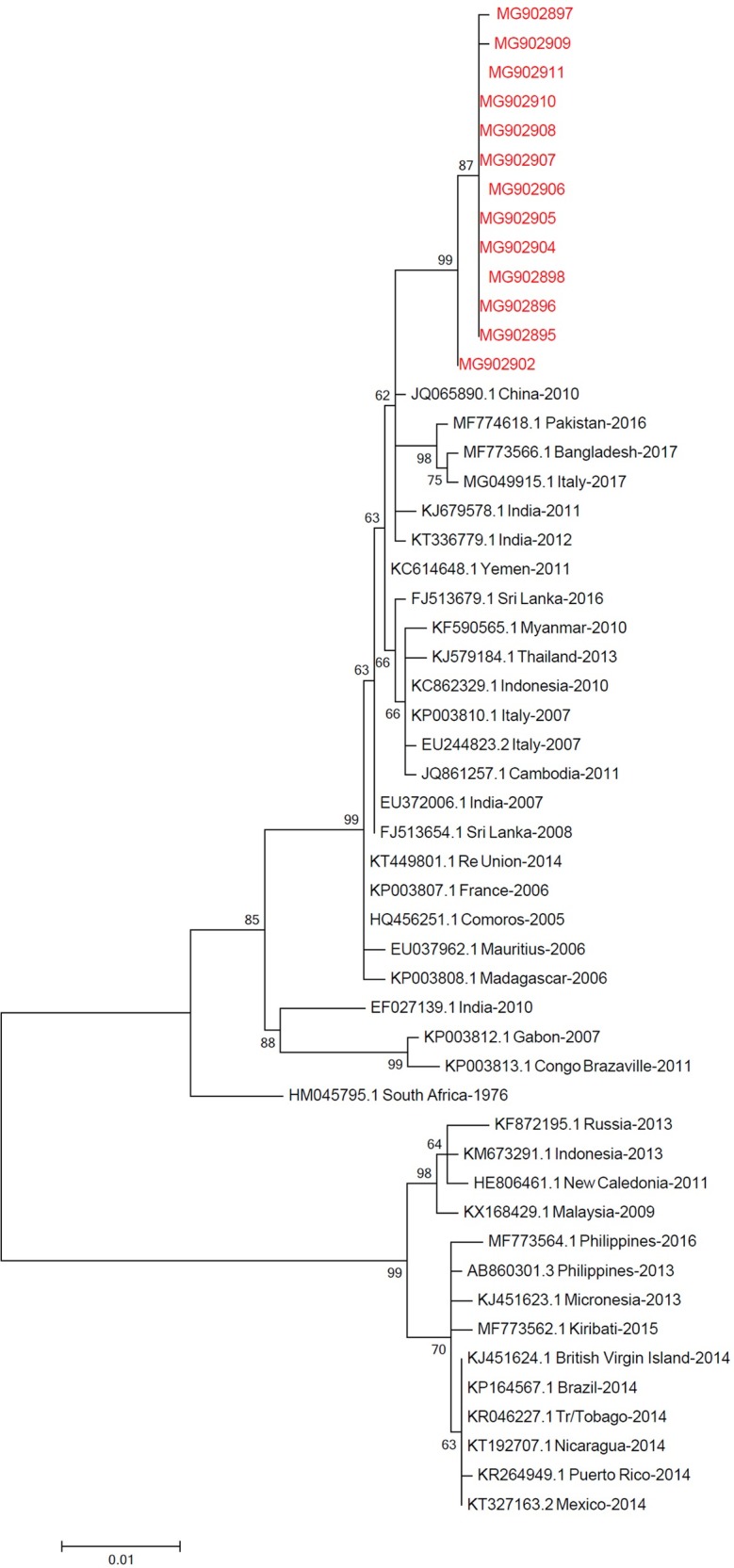
Phylogenetic relationships of Chikungunya virus strains. The tree was inferred in MEGA version 7 using 1138 base pair data set that encompasses the capsid protein. The analysis involved 52 nucleotide sequences. The Kenyan dataset is in the red typeface.

Sequence analysis of 13 isolates from Mandera associated with this outbreak revealed that though the viruses were closely related to those from China isolated in 2010, they were clearly distinct, but with very little variation between them (Fig 3).

### House, container and Breteau indices

From entomologic risk assessment, a total of 117 houses were inspected for *Ae*. *aegypti* mosquito immatures, of which 17 were positive, representing HI of 14.5%. In total, 279 wet containers were sampled indoors (n = 37; 13.3%) and outdoors (n = 242; 86.7%), of which 117 (41.9%); indoor n = 20 and outdoor n = 97 were positive for *Ae*. *aegypti* immatures, representing a BI of 17.1% and an overall CI of 41.9%. Overall, underground tanks were the most abundant (n = 84; 30.1%) and also with the highest positivity rate (n = 65; 77.4%), followed by barrels (n = 55) out of which 38.2% (n = 21) were positive. Although ground surface tanks were the second highest in terms of positivity, only four of them were sampled and we cannot fully conclude on their importance as preferred breeding sites relative to the other containers due to the numbers sampled. Tyres were third with 62.5% positivity rate (Table 1).

**Table 1:** The number of wet containers sampled indoors and outdoors and immature *Ae*. *aegypti* positivity rates.

	Indoor		outdoor		Total	
Container Type	Negative	Positive (%)	Negative	Positive (%)	Negative	Positive (%)
Underground tanks	0	0 (0)	19	65 (77.4)	19	65 (77.4)
Metal/plastic* barrels	8	14 (63.6)	26	7 (21.2)	34	21 (38.2)
Jerry cans	4	5 (55.6)	23	4 (14.8)	27	9 (25)
Buckets	5	1 (20)	32	2 (5.9)	37	3 (7.5)
Jars	0	0 (0)	6	0 (0)	6	0 (0)
Ground surface tanks	0	0 (0)	1	3 (75)	1	3 (75)
Plastic containers	0	0 (0)	9	5 (35.7)	9	5 (35.7)
Tyres	0	0 (0)	6	10 (62.5)	6	10 (62.5)
Cooking pans	0	0 (0)	4	1 (25)	4	1 (25)
Small cans	0	0 (0)	18	0 (0)	18	0 (0)
Potted plants	0	0 (0)	1	0 (0)	1	0 (0)
**Total**	17	20 (54.1)	145	97 (66.9)	162	117 (72.2)

Percentages of positive containers are indicated in parenthesis within the table

* = 50–200 liters capacity

## Discussion

The Kenyan coast was the epicenter of a major chikungunya outbreak in 2004 that resulted in thousands of human cases in Lamu and the subsequent spread to several islands in the Indian Ocean infecting millions of people [11, 14]. The geographical range of chikungunya continues to expand rapidly from Sub-Saharan Africa where it’s endemic to parts of Europe and Americas, facilitated by increased globalization and spread of the pathogen and its vectors. From 2004–2016, no acute cases of chikungunya were detected. However evidence of chikungunya circulation continued to be generated via serological survey studies targeting coastal and western regions of Kenya. In these studies, prevalence rates ranged from 0.97–42%, depending on the populations and regions sampled, with areas along the Kenyan coast recording the highest [27–31]. The outbreak being reported here mainly affected northeastern Kenya with Mandera town recording the most cases. This is the first time that chikungunya has been documented in northeastern Kenya. Chikungunya was first detected by RT-PCR in samples sent from the Bula-Hawa region of Somalia to Kenya in February 2016, with no case detected in Kenya in this period. In May 2016, the first cases of chikungunya were detected in Mandera which also contributed the highest percentage (89.6% of the total samples (126) tested from Kenya. Imported cases from Somalia were also detected in Italy in June 2016 [32]. Prior to 2016, there had been poor documentation of chikungunya circulation in Somalia in particular and the horn of Africa in general. Available information was based on technical and newspaper reports by various United Nations agencies and governments [32]. It is not clear what triggered the cases in Somalia, but the presence of *Ae*. *aegypti* mosquito species in towns along its coast and ideal environmental conditions are potential risk factors for chikungunya transmission [33]. Mandera is a town located in an arid area with erratic rainfall, water for routine household use is scarce and long term storage in various container types is common practice. In this case, most people store water in underground tanks which may therefore serve as permanent breeding sites and continuous source of *Ae*. *aegypti* mosquitoes as it is very difficult to completely drain out the water [34].

Entomological data shows that underground tanks were present almost in all homesteads and over 77% of them were infested with *Ae*. *aegypti* immatures. These containers, which are preferred for water storage and preservation, provide favorable breeding conditions for *Ae*. *aegypti* mosquitoes throughout the year regardless of the season. This is well supported by the high immature indices (HI = 14.5%; CI = 41.9%; BI = 17.1%) which are useful in indicating the breeding sites for *Ae*. *aegypti* and were above the WHO thresholds for initiating and sustaining an outbreak. These indices were also indicative of high *Ae*. *aegypti* densities that was the reason for widespread transmission of chikungunya. CHIKV incidence in the collected immature *Ae*. *aegypti* mosquitoes was not determined, nor were adult mosquitoes collected due to the logistics of transporting dry ice and dry shippers from Nairobi to Mandera for adult collection, preservation and transportation.

The detection of chikungunya coincided with the detection of dengue cases caused mainly by dengue 2 and 3 which had been on the rise since 2011[17]. None of the samples tested positive for dengue by RT-PCR hence we did not obtain any information on the specific dengue serotype that was in circulation at the time of the outbreak. Although a positive dengue IgM result is an acceptable indicator of acute infection, anti-DENV IgM has been shown to persist for 2–3 months after initial infection [35] hence it is not clear if there was co-circulation of CHIKV and DENV during this time. However, based on previous dengue serotype circulation, the current detection of dengue IgM antibodies and chikungunya PCR and IgM in patient samples, suggests that these two viruses share the same geographical area in Mandera based on the presence of a common vector.

In Africa, co-infections have been documented in countries where both viruses are endemic [18, 36–38]. Occurring in similar geographic areas and sharing a common principal vector, CHIKV and DENV have the same urban epidemic transmission cycle [39]. Disease symptoms caused by the two viruses are also difficult to distinguish in absence of laboratory confirmation [40, 41]. This hampers the epidemiological understanding of these diseases which in turn affects patient management through the use of wrong interventions leading to negative influence on case outcomes [18]. The role of co-infections on disease severity is varied. While in some cases co-infections have been shown to impact on disease severity [42], no association has been observed in others [18]. Partial sequence analysis of isolates from this outbreak showed close relatedness to isolates from outbreaks in China in 2010 which were due to local and imported cases from India and Nigeria.

The Kenyan isolates shared similarity with the imported isolates in China which had a 90% nucleotide identity related to the ECSA within Indian Ocean lineage [43]. Detection of similar isolates over wide geographical areas shows how fast and wide impacts can be felt from the countries where the outbreaks originally started [13]. In addition, imported cases are associated with risk of secondary transmission where conditions are ideal as it happened in Italy in 2007 where 205 cases were detected and the index case identified as a male from India who initiated the local transmission [44]. As global commerce expands cases of importation into countries throughout Europe, Asia and North America far from the original outbreak points have been documented [10, 15, 16].

As the geographical range of chikungunya continues to expand in Kenya, awareness creation among health care workers on rapid case detection, referral of samples to appropriate testing facilities for confirmation will be paramount in establishing the burden of disease and providing mitigation measures.

## Conclusion

We report on the first documented evidence of active chikungunya infection in Northern Kenya. The geographical range of chikungunya continues to expand and the evidence provided suggests that chikungunya and dengue share the same eco-system in Northern Kenya. Due to this overlap, there is a need to have diagnostic algorithms that are able to identify and distinguish infections caused by the two viruses. In addition, sustained entomological surveillance and vector control programs targeting the most productive containers are needed to detect these two viruses early and prevent possible outbreaks. Detection and early warning systems need to be strengthened in the horn of Africa to determine the true range of chikungunya and its, overlap with dengue. This will also establish the circulating virus genotypes and provide information on threat levels, risk for further spread outside its endemic areas, and enhance preparedness efforts in the region and globally.

## Disclaimer

Material has been reviewed by the Walter Reed Army Institute of Research. There is no objection to its presentation and/or publication. The opinions or assertions contained herein are the private views of the author, and are not to be construed as official, or as reflecting true views of the Department of the Army or the Department of Defense. The investigators have adhered to the policies for protection of human subjects as prescribed in AR 70–25.

## Supporting information

S1 File CHIKV sequences. **S**equences of 13 samples from Mandera; Kenya submitted to the gene bank and analyzed to determine their phylogenetic relationships to other Chikungunya virus strains.
(ZIP)
